# British Medical Association: The Presidential Address

**Published:** 1895-08-03

**Authors:** 


### Medical Progress and Hospital Clinics.

[*The Editor will be glad to receive offers of co-operation and contributions from members of the profession. All letters should be addressed to* The Editor, The Lodge, Porchester Square, London, W.]

### BRITISH MEDICAL ASSOCIATION.

### THE PRESIDENTIAL ADDRESS.

Sir Russell Reynolds, in his Presidential Address, drew attention to the great advances which had been made in every department of medicine since the last meeting of the British Medical Association in London twenty-two years ago, alluding especially to the vast strides which have been made in the elucidation both of the structure and function of different organs, in the development of bacteriology as a special science, and in the opening out of a new field of therapeutics in the serum treatment of disease. One matter which must be borne in mind was the power of "living" things, both for good and for evil, which seemed to him to be the most striking fact brought out by modern physiological, pathological, and therapeutical research. Hitherto we had received help from the mineral and inorganic world, but now our help and hope must come from organised products and still living agencies; and for the investigation of these agencies the building on the Thames Embankment commonly known as the Examination Hall, in which so many of the meetings of the Association were to be held, had become the centre of a very hive of industrious work. Medical teaching also had made great advances, and great improvements had been introduced into the common methods of instruction. Much of the old-fashioned, often dreary, "lecture" of an hour's duration had passed away, and in its place were found a demonstration, an "object-lesson," a conversation, or an examination. These were more useful, and, therefore, more attractive to the student, although often, no doubt, more trying to the teachers.

Allusion was made to the enormous increase which had taken place during recent years in the mass of medical literature. Not only in the many sections of the annual meeting of the Association, but in the numerous metropolitan and provincial societies, cases were recorded and discussions took place, which were published in magazines, "Proceedings," and other appropriate journals. It would, of course, be too much to say that the merit of this literary product of so many minds was of the highest order, but the mass of material was enormous, and, even although much of it might not be new or even true, the bringing together of recent facts, and the friction of mind with mind which took place in meetings of such societies was useful in separating the real and substantial from the imaginary or accidental. He would, however, draw attention to the fact that, great as had been the advances in our knowledge of diseases, their anatomy, pathology, and inter-relations, in regard to nosology and nomenclature there was little of which we could be proud. He especially ridiculed the custom of naming diseases after their first describers' names. We already had not only Bright's disease, Addison's, and Graves's, but a host of conditions, often indeterminate in character, going by the name of Friedreich, Raynaud, Hodgkin, and others, until there was a fair prospect of every distinguished pathologist or clinical investigator being handed down to posterity as a disease, the name of which would convey no semblance of a meaning as to its nature. Still more to be deplored was the habit of dealing in the same way with small groups of symptoms which were not honoured by being spoken of as separate diseases, but still had the names of their observers tied on to them, so that we had Cheyne-Stokes' breathing, Corrigan's pulse, von Graefe's sign, somebody else's phenomenon, and a multitude of other like phrases, which were used to the great confusion of the student and to his real and permanent injury. He continued, "Of course, to give the proper name to a disease is to place it where it should be, nosologically, 'at home,' and understood; but medicine is not a science yet sufficiently 'ordinated' for this thing to be done; we can, as yet, 'predict' with certainty but little, and so we grope about, and 'gather dust and chaff,' with little chance or hope of turning them to value; and instead of making this the matter of most serious investigation, and finding the true relation between the new 'group of symptoms,' and those others which form the central facts, the backbone, and whole skeleton of pathology, we lay hold of the first piece of red tape ready to our hand, tie up the group of symptoms in a bundle, and label it Brown, Jones, or Robinson's disease! A suggestion was made, not long ago, by one of our most able and energetic thinkers, that some apparently new diseases might perhaps with advantage be named, provisionally, after the patients who exhibited them. This certainly had the merit of originality, as it struck me at the time I heard it, but in looking at my case-books I found that already I had several times adopted it, in so far as to say, 'Case X Y Z resembling in particular features, that of A B C,' or some other very familiar patient. But nothing could be worse for scientific medicine than any such retrograde proceeding. If there be any danger of nomenclature resolving itself into a catalogue of the names of distinguished pathologists, as has been suggested, it would be a still more terrible disaster if a differentiation should be carried still further, on this dual system of nomenclature, and that the student should have to be examined in his knowledge of the 'George Brown variety of Robinson's disease,' or the 'Max Schott's deviation from von Hopner's sign.'"

Referring to the more recent discoveries in regard to immunisation by serum, he pointed out that the agents which acted most powerfully upon the human frame were all organic substances, the product of life. This knowledge was not new; its interest lay in the modern mode for its utilisation, for it would seem now that there was hardly a limit to what might be expected in the cure and prevention of disease by organic substances.

### The Address in Medicine.

Sir William Broadbent, in delivering the address in medicine, devoted himself mainly to sketching the growth of the art and science of medicine from the earliest times to its present position. Necessarily such an address was largely historical, but in his description of modern advances great stress was laid upon the importance of chemistry both in regard to pathology and therapeutics.

The Address in Surgery.

The address in surgery, delivered by Mr. Hutchinson, was a striking exposition of the advances which have been made in that art in recent times. Taking ovariotomy, lithotrity, and eye surgery as illustrations, he showed, however, that these advances have not altogether arisen from increased general knowledge, but have been largely due to a development of special skill among those who have been able to devote themselves largely to special departments, and he related how in his own case when he found specialists attaining greater success than he was able to achieve he had adopted the practice of handing over his cases for operation. Speaking of ovariotomy, he said, "I bore with such equanimity as I could the discovery that I could not compete with my friend in the ratio of successes obtained; and acting on the rule of conduct that I would never keep a patient in my own hands if I believed that someone else could do what was needed with greater prospect of success, I gave up doing ovariotomies both in public and private, and used to transfer my patients from the London to the Samaritan Hospital." And he asks, "Are there not also many other procedures which stand on precisely the same footing? Would anyone allow an inexperienced surgeon to extract a gall stone when the services of one who had done many such operations were at hand, or entrust the removal of laryngeal growths to any surgeon, however skilled, who was not specially trained as regards that operation?" Mr. Hutchinson suggested that if we could get the gross statistics of ovariotomy and lithotrity for the whole kingdom the ratio of success would fall lamentably short of that habitually secured by our best specialists. He spoke, however, of specialists rather than of special hospitals, for it was quite possible that the latter might, when the staff was large in proportion to the number of beds, cease to afford the necessary individual experience. It is a personal specialist which Mr. Hutchinson appeared to think so necessary. He considered that not only did these personal specialists develop remarkable skill themselves, but they almost invariably succeeded as teachers in imparting their skill to others, and he broadly laid it down that a man who thoroughly understood his subject in all its details could treat it with an efficiency and ease which were wholly unattainable by others. To encourage specialism in many branches of operative surgery was, then, he could not but think, in consonance with common sense and common humanity. There were operations which anyone decently trained and clever with his hands could do; there were others which are required so rarely that no one could possibly obtain much personal experience in their performance. But there were others which were good in themselves, and based on sound rules of surgery, but which were yet not for every surgeon's knife, and respecting which he thought that the canon of surgery should be that unless the skilled diagnostician and the skilled manipulator were at hand, it was to the patient's advantage that they should be avoided altogether. In this category he placed, along with many others, operations on the gall bladder and kidney, those for cancer of the bowel, laparotomies for obstructions, and trephining for abscess or tumours of the brain. In most of these the patient had some chance of recovery if he were left alone, whereas he had almost none if an unskilled operator took the knife in hand.

